# Tumor Microenvironment-Responsive 6-Mercaptopurine-Releasing Injectable Hydrogel for Colon Cancer Treatment

**DOI:** 10.3390/gels9040319

**Published:** 2023-04-10

**Authors:** Sungjun Kim, Wonjeong Lee, Heewon Park, Kyobum Kim

**Affiliations:** Department of Chemical & Biochemical Engineering, Dongguk University, 30, Pildong-ro 1-gil, Jung-gu, Seoul 22012, Republic of Korea; sungjun.kim@dgu.ac.kr (S.K.); jeong99@dgu.ac.kr (W.L.); hana4339@gmail.com (H.P.)

**Keywords:** hydrogel, colon cancer, 6-mercaptopurine, drug delivery system, anticancer

## Abstract

Colon cancer is a significant health concern. The development of effective drug delivery systems is critical for improving treatment outcomes. In this study, we developed a drug delivery system for colon cancer treatment by embedding 6-mercaptopurine (6-MP), an anticancer drug, in a thiolated gelatin/polyethylene glycol diacrylate hydrogel (6MP-GPGel). The 6MP-GPGel continuously released 6-MP, the anticancer drug. The release rate of 6-MP was further accelerated in an acidic or glutathione environment that mimicked a tumor microenvironment. In addition, when pure 6-MP was used for treatment, cancer cells proliferated again from day 5, whereas a continuous supply of 6-MP from the 6MP-GPGel continuously suppressed the survival rate of cancer cells. In conclusion, our study demonstrates that embedding 6-MP in a hydrogel formulation can improve the efficacy of colon cancer treatment and may serve as a promising minimally invasive and localized drug delivery system for future development.

## 1. Introduction

Colon cancer, also known as colorectal cancer, is a type of cancer that develops in the colon or rectum. It is the third most common cancer and the second leading cause of cancer-related deaths in the United States [1,2]. Treatment for colon cancer typically involves a combination of surgery, chemotherapy, and radiation therapy. However, these therapies have side effects and limited therapeutic efficacy [3]. For instance, depending on the location of the cancer, a partial colectomy might be performed to remove a portion of the colon, or a rectal resection is performed to remove the rectum. Radiation therapy can cause damage to healthy tissues surrounding the tumor, leading to side effects, such as fatigue, skin irritation, and damage to organs. Moreover, chemotherapy for colon cancer could cause significant side effects, including liver toxicities, gastrointestinal toxicities, and hematologic disorders [4,5].

A drug delivery system that responds to and localizes in the tumor microenvironment (TME) can be a technology that maximizes the efficacy of anticancer treatment while minimizing the side effects of existing anticancer drugs [6,7]. Recently, hydrogels have attracted a lot of interest in the field of anticancer therapy due to their ability to encapsulate drugs, peptides, and other therapeutic agents and deliver them to targeted sites in a controlled and sustained manner [8,9,10,11]. One of the key advantages of using hydrogels for cancer therapy is localized drug delivery to the tumor site, which can reduce the side effects associated with systemic drug administration. Additionally, hydrogels could be designed to respond to different stimuli, such as changes in pH, temperature, and enzyme activity, which allows for the targeted release of therapeutic agents in response to specific conditions in the TME. Among various chemical bonds, the disulfide bond is an attractive candidate that can be dissociated in response to GSH or an acidic pH [12,13,14,15,16,17].

**Figure 1:**
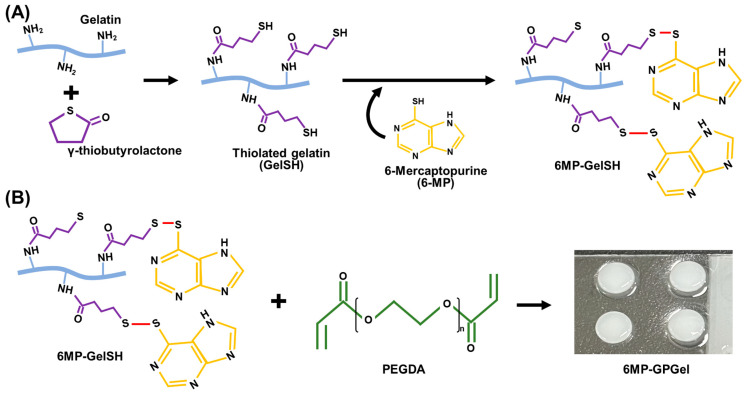
A schematic illustration of (**A**) the synthesis of thiolated gelatin and disulfide bond-mediated 6-MP conjugation, and (**B**), the fabrication of 6MP-GPGel.

In this study, we developed an anticancer drug (6-mercaptopurine, 6MP), an embedded composite hydrogel, consisting of thiolated gelatin (GelSH) and poly(ethylene glycol) diacrylate (PEGDA). Various natural and synthetic polymers have been utilized for hydrogel fabrication. Gelatin, a denatured biomacromolecule from collagen, possesses desirable physicochemical properties, such as biocompatibility, low antigenicity, biodegradability, and cell attachability [18,19]. Additionally, gelatin is recognized by the FDA as generally safe for consumption (GRAS) [20]. Despite its benefits, gelatin has limitations, such as low mechanical strength, low thermal stability, and rapid water degradation, which hinder its use in versatile tissue engineering applications [21,22]. To overcome these issues, gelatin molecules can be chemically modified to increase crosslinking density or incorporated into interpenetrating polymer networks (IPNs) with complementary substances. Thiolated polymers have been extensively employed for hydrogel design due to the high reactivity of the sulfhydryl moiety [23]. In the fabrication of IPN hydrogels, GelSH was combined with a synthetic PEGDA polymer, which offers excellent controllability. This GelSH/PEGDA hydrogel (GPGel) can be used as an implantable scaffold for various tissue engineering applications including bone and osteochondral tissues [18,24,25]. A series of studies have demonstrated, in vitro/in vivo, the long-term stability of GPGel. Moreover, GPGel exhibits non-immunogenicity and excellent biocompatibility. 6-MP is a well-known anticancer drug that can induce apoptosis by interfering with DNA replication and RNA transcription [26,27]. In this regard, 6-MP was conjugated with GelSH (6MP-GelSH) through a disulfide bond (Figure 1A) and then gelated with PEGDA (Figure 1B). Hence, our 6-MP-embedded GPGel (6MP-GPGel) could be used as an effective scaffold that is injectable, with high stability and the ability to release drugs in response to the TME.

Herein, we evaluated the anticancer function of 6MP-GPGel as an injectable and implantable composite hydrogel scaffold. It was found that 6-MP was highly released from 6MP-GPGel in response to the TME conditions, especially acidic pH and high GSH environments. Consequently, 6-MP released from 6MP-GPGel effectively inhibits colon cancer cells (i.e., HCT-116). The objectives of the current study were to investigate the (1) sensitivity of colon cancer cells to 6-MP, (2) TME-responsive 6-MP release, and (3) anticancer activity of 6MP-GPGel.

## 2. Results and Discussion

### 2.1. Sensitivity of Colon Cancer to 6-Mercaptopurine

**Figure 2:**
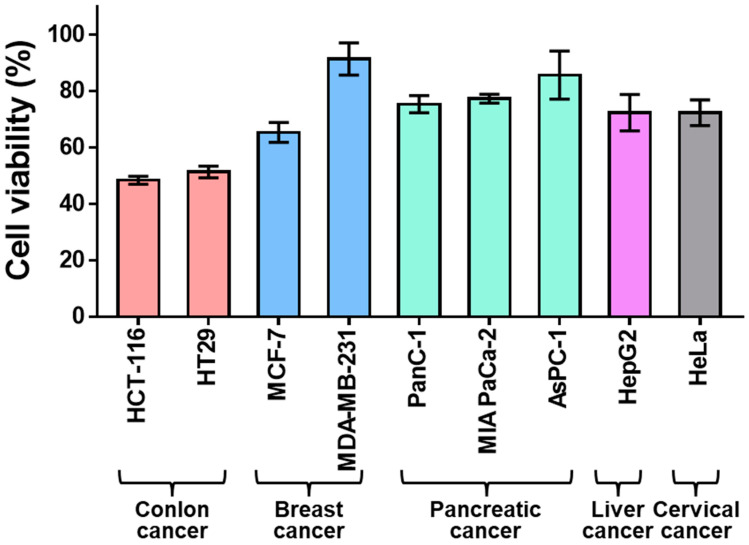
Anticancer efficacy of 6-MP (50 μg/mL) against various cancer cells including colon cancer, breast cancer, pancreatic cancer, liver cancer, and cervical cancer cells.

**Figure 3:**
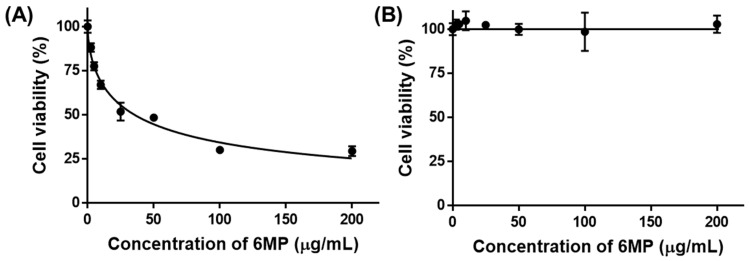
Viability of (**A**) HCT-116 cells and (**B**) fibroblasts after treatment with different concentrations of 6-MP.

TRAIL 6-MP is an analog of thiopurine and it is a well-known anticancer drug that can inhibit the growth of tumor cells by interfering with purine synthesis, which is closely related to DNA and RNA biosynthesis [28]. In this study, nine types of cancer cells including colon cancer, breast cancer, pancreatic cancer, liver cancer, and cervical cancer were treated with 6-MP to select cancer cell lines sensitive to 6-MP. As a result, colorectal cancer cells, HCT-116 and HT29, showed significantly lower cell viability compared to breast cancers (MCF-7 and MDA-MB-231), pancreatic cancers (PanC-1, MIA PaCa-2, and AsPC-1), liver cancer (HepG2), and cervical cancer (HeLa) (Figure 2). These results demonstrated that colon cancers are sensitive to 6-MP. In addition, slightly lower cell viability was found for HCT-116 (48.4%) compared to that of HT29 (51.3%). Therefore, we designated HCT-116 as a major target for cancer. The viability of HCT-116 cells was 6-MP concentration-dependent. The IC_50_ value of 6-MP for HCT-116 cells was 36.1 μg/mL (Figure 3). Notably, no toxicity was observed for fibroblasts in a normal cell model after treatment with 6-MP at concentrations of up to 200 μg/mL. Consequently, 6-MP could be used as an excellent anticancer drug candidate that can sensitively kill colon cancer without any toxicity to normal cells.

### 2.2. Characterization of Hydrogel

**Figure 4:**
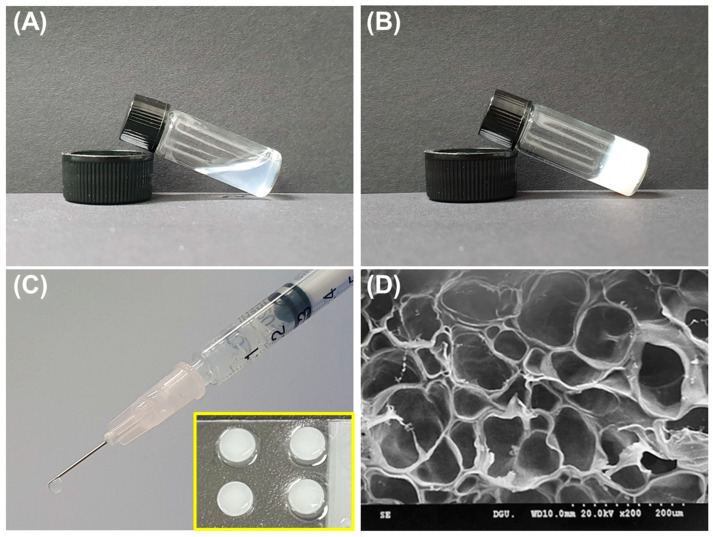
Characterization of 6MP-GPGel. (**A**,**B**) Sol–gel phase transition of hydrogels; (**C**) macroscopic image of hydrogel injection using a syringe. Yellow box showing hydrogels fabricated using a Teflon mold; and (**D**) SEM image of cross-sectioned 6MP-GPGel.

In this study, we conjugated 6-MP to GelSH via disulfide bonds (Figure 1A), and the success of the conjugation was confirmed through UV spectroscopy. Specifically, GelSH did not exhibit a peak around 320 nm, whereas 6MP-GelSH showed a distinctive peak at that wavelength (Figure S1). These results demonstrated that 6-MP was effectively conjugated to GelSH. Then, hydrogels were fabricated by mixing 6MP-GelSH and PEGDA. Gelatin has desirable intrinsic physicochemical properties such as excellent biocompatibility, biodegradability, and low antigenicity. It is well known as a suitable hydrogel material [29,30]. However, hydrogels made only from gelatin often exhibit disadvantages, such as poor mechanical properties, low thermal stability, and instantaneous dissolution in water, which often cause failure in tissue engineering applications [21,22]. Fabricating hydrogels in combined use with PEGDA can be a way to overcome these problems. The resulting GPGel shows improved physical and chemical fragility of the component by increasing its mechanical strength through chain-wide polymerization [31,32]. Here, gelation by sol–gel phase transition of the 6MP-GPGel was evaluated using a vial tilting method (Figure 4A,B). The prepared sol solution was injectable. It could be cast into a desired shape according to the mold (Figure 4C). In addition, we used SEM to observe the 3D interconnected porous structure of 6MP-GPGel, which revealed a pore size of approximately 81.8 ± 30.2 μm (Figure S2). The porous architecture of hydrogels has a significant impact on various mechanical properties, including biodegradability and Young’s modulus. In a previous study, we investigated the structural–physical relationship of GPGel and synthesized GPGels with various ratios of PEGDA and GelSH [18]. Our results showed that GPGel with a smaller pore size exhibited improved mechanical properties, such as slower degradation and higher Young’s modulus. Therefore, by adjusting the content of GelSH and PEGDA, it is possible to achieve desired properties and customize GPGels for specific applications.

### 2.3. Release of 6-MP from 6MP-GPGel

**Figure 5:**
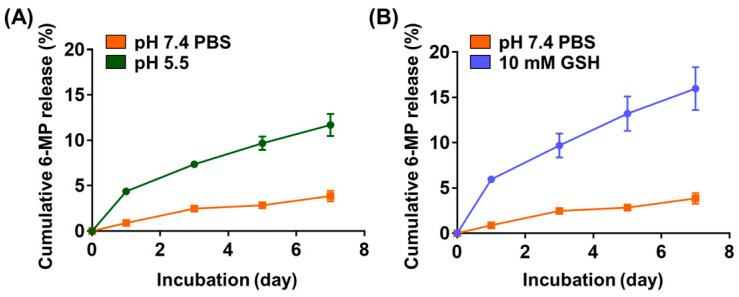
% cumulative 6-MP release profile at (**A**) different pH and (**B**) glutathione concentrations.

**Figure 6:**
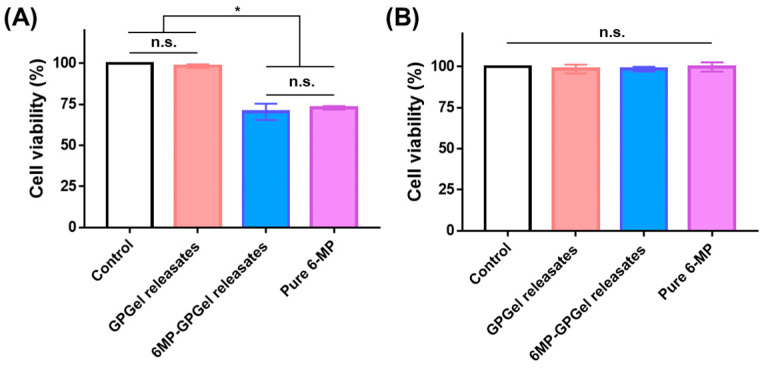
Biological anticancer activity and biocompatibility of 6-MP released from 6MP-GPGel to (**A**) HCT-116 cells and (**B**) fibroblasts. (*) indicates that experimental groups are statistically different from each other. (n.s.) indicates that experimental groups are not significantly different.

6MP-GPGels containing 10 μg of 6-MP were fabricated, and the release profile of 6-MP was evaluated in TME-mimicking environments [33]. Under PBS conditions, a sustained 6-MP release profile was observed, and 3.9% of cumulative release was recorded over 7 days (Figure 5A,B). In contrast, a burst 6-MP release profile was observed in TME-mimicking conditions, such as an acidic pH (pH 5.5) (Figure 5A) and higher GSH concentration (10 mM) (Figure 5B). Specifically, we observed 11.7% and 16.0% of cumulative release for 7 days at pH 5.5 and 10 mM GSH conditions, respectively. These results might be caused by the acidic pH and various reducing activities of GSH. Tumor tissues exhibit exclusive cellular microenvironments (e.g., acidic, enzymatic, and reducing environments) due to their unique physiological properties [34,35]. In a GSH-reducing environment, polymeric materials with disulfide bonds can depolymerize at a faster rate than other types of redox-reactive carriers. Moreover, the pH of the TME ranges from 5.5–7.0 [36]. Therefore, in this study, we conducted a 6-MP release experiment in an environment with a pH of 5.5, which could significantly accelerate the release of 6-MP. Accordingly, a reducing environment by pH could also accelerate drug release [12,37]. In addition, HCT-116 cells were treated with 6-MP released from 6MP-GPGel to estimate the maintenance of the anticancer efficacy of 6-MP (Figure 6A). As a result, 6-MP released from 6MP-GPGel showed anticancer activity similar to that of pure 6-MP at the same concentration. These results demonstrated that the series of processes in which 6-MP was embedded and released in our hydrogel system did not affect the biochemical functionality of 6-MP. Additionally, in vitro biocompatibility tests were performed according to the International Organization for Standardization 10993-5 and previous related studies [18,38,39]. Treatment with 6-MP-free GpGel releasates showed no toxicity to HCT-116 cells (Figure 6A) or fibroblasts (Figure 6B). In addition, the 6MP-GPGel releasates failed to kill fibroblasts as 6-MP did not induce any toxicity in fibroblasts, as previously demonstrated (Figure 3B). The biosafety of GPGel-based hydrogel systems has been demonstrated in a series of in vitro and in vivo studies [18,24,25]. The cytocompatibility was assessed in vitro using two methods: (1) treatment of cell cultures with GPGel releasates [18], and (2) encapsulation of cells within the hydrogel [24]. No toxicity was observed when fibroblasts or HaCaT cells were treated with releasates of GPGel prepared using various combinations of GelSH, PEGDA, and initiator (APS or TEMED) [18]. Additionally, adipose-derived mesenchymal stem cells were encapsulated within GPGel and cultured for 14 days, with only green-stained cells (i.e., live cells) observed [24]. In vivo biocompatibility was confirmed by implanting GPGel subcutaneously in mice for 3, 7, and 15 days. Tissue sections harvested at each time point were stained with a CD68 macrophage marker for inflammation analysis, with the control PEGDA hydrogel group exhibiting a severe inflammatory response, while the GPGel-induced group only displayed a mild response [24]. Furthermore, when GPGel was implanted into mouse calvarial bone [25] and osteochondral defects [24], no necrosis or toxicity of surrounding tissue was observed. Therefore, our smart hydrogel system, 6MP-GPGel, could achieve effective anticancer activity by rapidly releasing drugs under pH and GSH conditions in the TME with excellent biocompatibility.

### 2.4. Anticancer Efficacy of 6MP-GPGel

**Figure 7:**
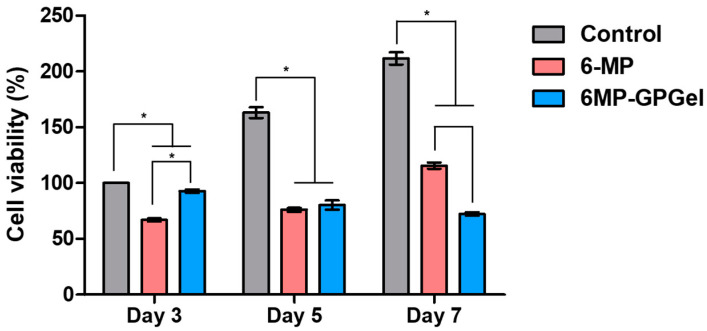
In vitro anticancer effects of 6MP-GPGel on colon cancer HCT-116 cells. (*) indicates that experimental groups are statistically different from each other.

Colon cancer recurrence is a risk factor that occurs in 30–50% of patients after standard chemotherapy [40]. Therefore, it is important to not only drastically reduce cancer at the beginning of treatment but also to continuously suppress colon cancer. In this study, the viability of HCT-116 cells was examined for 7 days (Figure 7). In the control HCT-116 group, cells continued to proliferate over time. The cell viability decreased to 66.9% on day 3, but increased to 76.1% and 115.5% on days 5 and 7, respectively, in the pure 6-MP-treated group. Inactivation of 6-MP often causes failure of continuous treatment. Thus, it is necessary to develop a delivery system capable of stably supplying the drug [41]. The viability of HCT-116 cells cultured with 6MP-GPGel decreased over time to 92.7%, 83.7%, and 72.3% at 3, 5, and 7 days, respectively. These results were attributed to the continuous supply of 6-MP in response to the TME (Figure 5) while maintaining the biochemical activity of 6-MP (Figure 6) in the 6MP-GPGel. Various carriers have been developed to deliver 6-MP in recent decades. For instance, Nezhad-Mokhtari et al. developed multi-stimuli-responsive polymeric nanogels [42]. These nanogels were linked to 6-MP through a disulfide bond and released 6-MP in response to GSH and an acidic environment (pH 5.3). The nanogels were effectively uptaken by MCF-7 cells and induced the apoptosis of cancer cells. Additionally, Jakubowski et al. developed zinc zeolite particles loaded with 6-MP, which demonstrated a significant improvement in anticancer efficacy compared to standard bolus 6-MP treatment [43]. This drug delivery system, based on particles, can effectively accumulate in tumor tissue through the enhanced permeability and retention effect and the binding of various targeting molecules. However, the challenge of overcoming their accumulation primarily in the liver and kidney upon intravenous injection still remains. In contrast, our hydrogel-based 6-MP delivery system can be injected and localized around the cancer, preventing accumulation in other organs in the body. To the best of our knowledge, this is the first study to develop a localized hydrogel-based 6-MP delivery system that is recognized by the tumor microenvironment. Additionally, in our previous studies, we demonstrated a significant increase in the therapeutic efficacy of GPGel by encapsulating coacervate, a protein drug delivery carrier, and/or therapeutic cells (ADSCs) into the hydrogel [24,25]. The encapsulation with coacervate was found to further improve long-term treatment efficiency compared to direct drug loading into GPGels. Coacervate is a self-assembling microdroplet that has been used as a versatile protein therapeutic drug delivery system [44,45,46]. When a protein therapeutic agent for anticancer treatment was loaded into coacervate, it protected the internal protein from the harsh external environment and released the therapeutic agent in response to the TME [6]. For instance, the rapid release of TRAIL was observed when it was loaded into coacervate in the highly ionic environment of the TME. This allowed the TRAIL-loaded coacervate to effectively inhibit the recurrence of colon cancer [3]. Loading additional anticancer drugs into these coacervates and embedding them in 6MP-GPGel has the potential to further maximize their anticancer functionality. Taken together, these results suggest that the noninvasive local administration of 6MP-GPGel with excellent biocompatibility could effectively induce colon cancer cell death and suppress recurrence.

## 3. Conclusions

In this study, the 6MP-GPGel was manufactured and the anticancer efficacy was evaluated. 6MP was more sensitive to colon cancer than breast cancer, pancreatic cancer, liver cancer, and uterine cancer. It induced colon cancer cell death. In particular, among two types of colon cancer cells, HCT-116 and HT29, HCT-116 showed slightly higher sensitivity. After that, 6-MP was conjugated with GelSH to produce an injectable composite IPN hydrogel. In addition, 6MP-GPGel exhibited (1) excellent biocompatibility, (2) a sustained 6-MP release profile, (3) a TME-responsive 6-MP release, and (4) retained anticancer functionality of the released 6-MP. The treatment of HCT-116 cells with 6-MP treatment resulted in early cancer cell death. However, inactivation over time led to the repopulation of cancer cells starting from day 5. In contrast, administering the 6MP-GPGel to HCT-116 cells inhibited cancer recurrence from day 5 and exhibited a more potent anticancer effect than 6-MP alone on day 7. The focus of developing anticancer drug delivery systems using 6-MP has mainly been on particle-based systems, which may accumulate not only in the target tumor tissue but also in various organs and tissues, resulting in potential toxicity and other problems. In contrast, the 6MP-GPGel developed in this study can induce the death of colon cancer by releasing 6-MP only in the localized area around the tumor tissue, thus minimizing the risk of unwanted tissue accumulation and associated toxicity. Therefore, our 6MP-GPGel system can serve as a novel protein drug delivery platform to inhibit the recurrence of residual cancer after cancer resection or chemotherapy by continuously supplying available 6-MP to the tumor. 

## 4. Materials and Methods

### 4.1. Materials

Gelatin, ethylenediaminetetraacetic acid (EDTA), γ-thiobutyrolactone, β-mercaptoethanol, imidazole, PEGDA, ammonium persulfate (APS), N,N,N,N′-tetramethylethylenediamine (TEMED), 6-MP, and Ellman’s reagent were purchased from Sigma–Aldrich. Dulbecco’s modified Eagle’s medium (DMEM), penicillin–streptomycin solution, and fetal bovine serum (FBS) were obtained from Corning. EZ-Cytox was bought from Daeil Lab Service.

### 4.2. Anticancer Efficacy of 6-Mercaptopurine

All types of cells were cultured in growth media consisting of DMEM (89% (*v*/*v*)), penicillin–streptomycin solution (1% (*v*/*v*)), and FBS (10% (*v*/*v*)). To evaluate the anticancer effect of 6-MP on colon cancer cells, various types of cancer cells including colon cancer (HCT-116 and HT29), breast cancer (MCF-7 and MDA-MB-231), pancreatic cancer (PANC-1, MIA PaCa-2, and AsPC-1), liver cancer (HepG2), and cervical cancer (HeLa) cells were treated with 6-MP. Cells were seeded at 10,000 cells/well into 96-well plates and incubated at 37 °C with 5% CO_2_ and 95% humidity for 24 h. The old medium was discarded and replaced with a fresh growth medium containing 50 μg/mL 6-MP. After 24 h of incubation, WST-1 reagent was prepared by mixing EZ-Cytox and growth medium at a 1:10 (*v*/*v*) ratio and was added to each well. WST-1 reagent-treated cells were incubated at 37 °C for 3 h. The optical density was then measured at 440 nm using microplate spectrophotometry. To investigate the concentration-dependent anticancer efficacy against colon cancer cells and the biosafety of 6-MP, HCT-116 cells and human fibroblasts were seeded at 10,000 cells/well on 96-well plates and incubated at 37 °C with 5% CO_2_ and 95% humidity for 24 h. Thereafter, the old medium was discarded and replaced with fresh growth medium containing 0–200 μg/mL 6-MP. After 24 h of incubation, the WST-1 assay was performed as described above.

### 4.3. Synthesis of Thiolated Gelatin

Gelatin was thiolated as described in our previous studies [18,24,25]. Briefly, 100 mL of 1% (*w*/*v*) gelatin solution was mixed with 1 mL of 1.85% (*w*/*v*) EDTA and 2 mL of 34% (*w*/*v*) imidazole. After 10 min of stirring, 840 μL of γ-thiobutyrolactone was added to the gelatin mixture and further reacted at 40 °C for 24 h. The reacted mixture was then dialyzed (molecular weight cutoff 14,000) against 0.2% (*v*/*v*) β-mercaptoethanol for 20 h and DW for the next 4 h. The produced GelSH was lyophilized and stored at −80 °C before use. The final thiol content determined by Ellman’s assay was 190.2 μmol/g.

### 4.4. Conjugation of 6-Mercaptopurine to Thiolated Gelatin

To conjugate 6-MP to GelSH, 30 mg of GelSH was dissolved in 3 mL of DW, and 5 mg of 6-MP was dissolved in 1 mL of DMSO. Subsequently, 6-MP was dropped into GelSH solution under stirring at 40 °C. The reactant was stirred for 24 h and dialyzed (molecular weight cutoff 14,000) against DW for 72 h. The final product (i.e., 6MP-GelSH) was lyophilized and stored at −80 °C before use. The UV spectra of GelSH and 6MP-GelSH were measured in the wavelength range of 280–500 nm. To measure 6-MP content in 6MP-GelSH, 5 mg of 6MP-GelSH was dissolved in 30 mL of HCl solution, and the optical density was measured at 320 nm using microplate spectrophotometry [47]. Here, GelSH (without 6-MP) was used to determine the background signal, and pure 6-MP was used as a standard. 

### 4.5. Fabrication of Drug-Embedded Composite Hydrogel

6MP-GelSH, APS, and TEMED were dissolved in DW at 5%, 4%, and 2% (*w*/*v*), respectively. PEGDA solutions were diluted to have a final concentration of 12.5% (*w*/*v*). 6MP-GelSH and PEGDA solutions were mixed first. Initiators (i.e., APS and TEMED) were added subsequently. For 2 mL of 6MP-GPGel, 1 mL of 6MP-GelSH, 0.8 mL of PEGDA, 0.1 mL of APS, and 0.1 mL of TEMED solutions were mixed. The mixed 6MP-GPGel was immediately transferred into a syringe and incubated at 37 °C for 30 min after injection.

### 4.6. Scanning Electron Microscopy

Three-dimensional porous structures of the 6MP-GPGel were observed with a scanning electron microscope (SEM). The prepared 6MP-GPGel solution was injected into a Teflon mold (8 mm in diameter and 2 mm in height) through a syringe and incubated at 37 °C for 30 min for gelation. Subsequently, the fabricated 6MP-GPGel was lyophilized until all solvents were completely removed. Dried hydrogels were coated in Pt by sputter (E-1010, HITACHI, Tokyo, Japan). The surface morphologies of the composite hydrogels were obtained using an SEM (S-3000 N, HITACHI) at a 20 kV accelerating voltage. Pore size was analyzed using ImageJ software (http://rsbweb.nih.gov/ij/, accessed on 6 March 2023, National Institutes of Health, Bethesda, MD, USA).

### 4.7. In Vitro 6-Mercaptopurine Release Profile

Hydrogels containing 10 μg of 6-MP were fabricated to determine the kinetics of 6-MP release from composite 6MP-GPGel hydrogels. Fabricated hydrogels were subsequently incubated with 1 mL of PBS at pH 5.5, or GSH solution (10 mM) solution on a shaker table at 100 rpm and 37 °C, for mimicking the TME. On days 1, 3, 5, and 7, the supernatant was collected after centrifugation and resuspended with each fresh solution. Released 6-MP was measured, as previously and above, and GPGel was used to determine the background signal. 

### 4.8. Anticancer Activity of Released 6-MP from Hydrogel

The bioactivity of the released 6-MP from 6MP-GPGel was estimated by comparison with same the amount of pure 6-MP. Briefly, 10,000 HCT-116 cells or fibroblasts were seeded onto 96-well plates and incubated at 37 °C with 5% CO_2_ and 95% humidity for 24 h. The old medium was then discarded and replaced with fresh growth medium containing hydrogel releasates (2.3 μg/mL collected 6-MP) or pure 6-MP. After 24 h of incubation, cell viability was measured using the WST-1 assay.

### 4.9. Anticancer Effect of Composite Hydrogel

To determine the anticancer effect of 6MP-GPGel, 100,000 HCT-116 cells were seeded into 24-well plates and incubated at 37 °C with 5% CO_2_ and 95% humidity for 24 h. The old medium was then changed to a fresh medium or 20 μM GSH-containing medium. The fabricated GPGel or 6MP-GPGel was deposited on the center of a Transwell^®^ insert and incubated with the prepared HCT-116 cells for 5 days. Cell viability was measured using the WST-1 assay.

### 4.10. Statistical Analysis

For quantitative data, all statistical analyses were conducted with GraphPad Prism 7.0 (GraphPad Software Inc., San Diego, CA, USA). Quantitative experiments were performed in triplicate and analyzed using one-way analysis of variance (ANOVA) and Tukey’s multiple-comparison test.

## Data Availability

Not applicable.

## Supplementary Materials

The following supporting information can be downloaded at: https://www.mdpi.com/article/10.3390/gels9040319/s1, Figure S1: UV spectra of GelSH and 6MP-GelSH.; Figure S2: Pore size distribution of 6MP-GPGel. Pore size was obtained from SEM images.
