# Supplementary material for: Tumor Microenvironment-Responsive 6-Mercaptopurine-Releasing Injectable Hydrogel for Colon Cancer Treatment
Source: Gels. 2023 Apr 10;9(4):319. doi: 10.3390/gels9040319 (PMC10138092; doi:10.3390/gels9040319)
Supplement: Supplementary file 1 [file gels-09-00319-s001.zip › gels-2314411-supplementary.pdf]

gels

Supplementary Materials

Tumor Microenvironment-Responsive 6-Mercaptopurine-Releasing Injectable Hydrogel for Colon Cancer Treatment

Sungjun Kim, Wonjeong Lee, Heewon Park and Kyobum Kim *

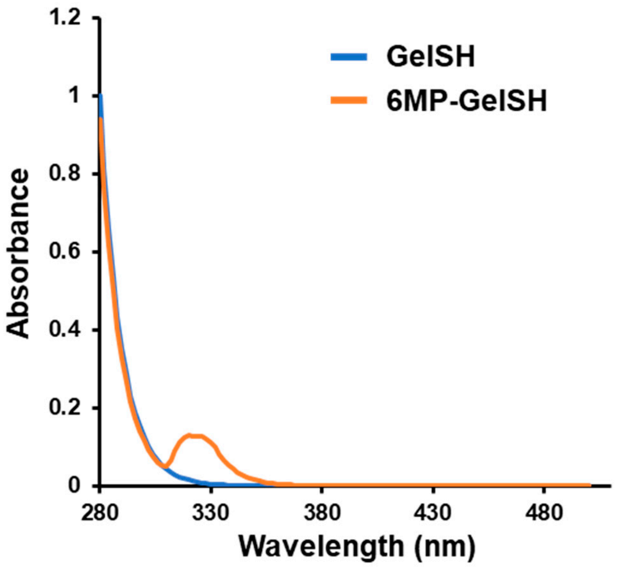

Figure S1. UV-spectra of GelSH and 6MP-GelSH.

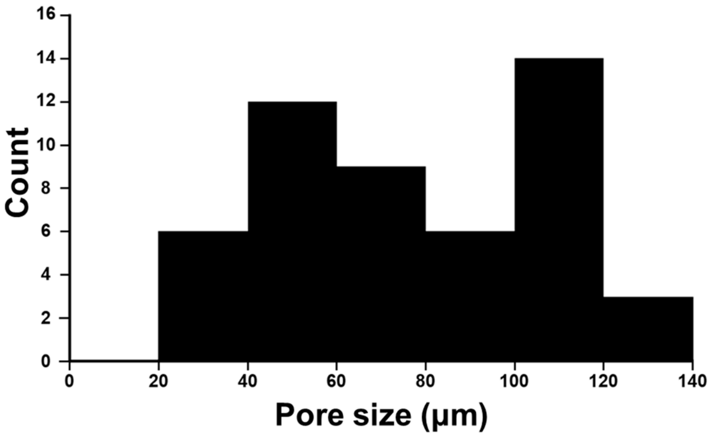

Figure S2. Pore size distribution of 6MP-GPGel. Pore size was obtained from SEM images.
